# Sliding metasurfaces: a two-in-one meta-device for frequency filtering and beam steering

**DOI:** 10.1038/s41377-026-02470-8

**Published:** 2026-10-16

**Authors:** Lei Xu, Mohsen Rahmani

**Affiliations:** Advanced Optics and Photonics Laboratory, Department of Engineering, School of Science & Technology, Nottingham Trent University, Nottingham, NG11 8NS UK

**Keywords:** Electronics, photonics and device physics, Photonic devices

## Abstract

Metasurfaces are considered the backbone of the ongoing photonics revolution to shape the electromagnetic waves on demand via a flat array of resonators. The beauty of metasurfaces lies in their ability to simultaneously control multiple waves. However, obtaining such multi-functionality often requires active components, such as active electronics and complex tuning mechanisms. A recent study demonstrates a fully passive architecture, composed of sliding metasurfaces that combines two distinct functionalities, i.e. frequency-selective filtering with wide-angle beam steering in a single platform. By replacing local phase reconfiguration with a simple geometric translation between complementary phase profiles, the work introduces a simple yet innovative route to a two-dimensional frequency-angle selection window. It opens new opportunities for passive wave-based information processing.

The ability to control where electromagnetic energy goes and which signals are allowed to pass lies at the heart of modern technologies, from wireless communications and radar systems to sensing and imaging platforms. Metasurfaces, ultrathin arrays of engineered subwavelength meta-atoms, have emerged as a versatile platform for manipulating electromagnetic waves, enabling functionalities ranging from beam steering and wavefront shaping to frequency-selective filtering within compact footprints^1–6^. However, those functions in general have largely evolved along separate paths. Spatial control is commonly achieved through engineered phase gradients that redirect electromagnetic energy^7^, whereas spectral control relies on resonant responses that selectively transmit, reflect, or absorb specific frequencies^8,9^. Nowadays, with increasing spectral congestion and functional integration, the ability to manipulate both the spatial and spectral properties of electromagnetic waves within a compact element has become an increasingly attractive goal. Most current tunable metasurfaces rely on active components such as PIN diodes, varactors, or phase-change materials to dynamically modify their electromagnetic response^10–12^. These approaches often come at the cost of increased hardware complexity, power consumption, fabrication challenges, and they in general, offer limited tuning ability and/or only work within specific frequency ranges. A simpler, more scalable route to multifunctional wave control is highly desirable and critical.

A recent work by H. Shi, W. Zhu, G. Hu and co-workers introduced an innovative approach to address this challenge^13^. Instead of dynamically modifying the electromagnetic response of individual meta-atoms using active elements, as in conventional tunable metasurfaces, this approach replaced local reconfiguration with a global geometric displacement: the relative lateral shift between two complementary metasurface layers. Through controlled sliding, such an approach can generate an effective phase gradient across the surface, rigidly translate the angular spectrum, and result in controlled beam deflection, allowing the transmitted beam to be steered without any active components or tuning of individual meta-atoms. Importantly, this operation preserves the intrinsic spectral filtering properties of the metasurface, enabling simultaneous control over frequency and propagation direction.

The key principle here can be interpreted as a translation of the angular spectrum in reciprocal space. The proposed unit cell of the metasurface consists of multiple stacked metallic and dielectric layers that form the resonant elements. These resonant elements in the structure generate effective inductive and capacitive responses, allowing the carefully engineered multilayer architecture to support a well-defined passband together with strong out-of-band suppression. By controlling the two independent geometric degrees of freedom of the 5th-layer split-ring resonator, the split orientation and the opening angle, the metasurface can provide stable phase biases within the transmission passband. As illustrated in Fig. 1, this dual functionality forms a frequency-angle selection window in which both the spectral content and propagation direction of the electromagnetic waves can be controlled within a single device in a continuously tunable fashion and enables a sharp bandpass response within the 26.5–29.5 GHz range, with out-of-band suppression exceeding 20 dB. When the two metasurface layers are perfectly aligned, their phase contributions cancel, producing a transmitted beam along the optical axis. As the layers are displaced relative to one another, a linear phase gradient naturally emerges, shifting the spectral centroid and consequently redirecting the transmitted beam, resulting in a compact platform capable of continuously tunable wide-angle beam steering. As a result, the metasurface maintains low insertion loss and stable phase relationships across the passband, ensuring consistent beamforming performance over a ±57° scanning range.

**Fig. 1:**
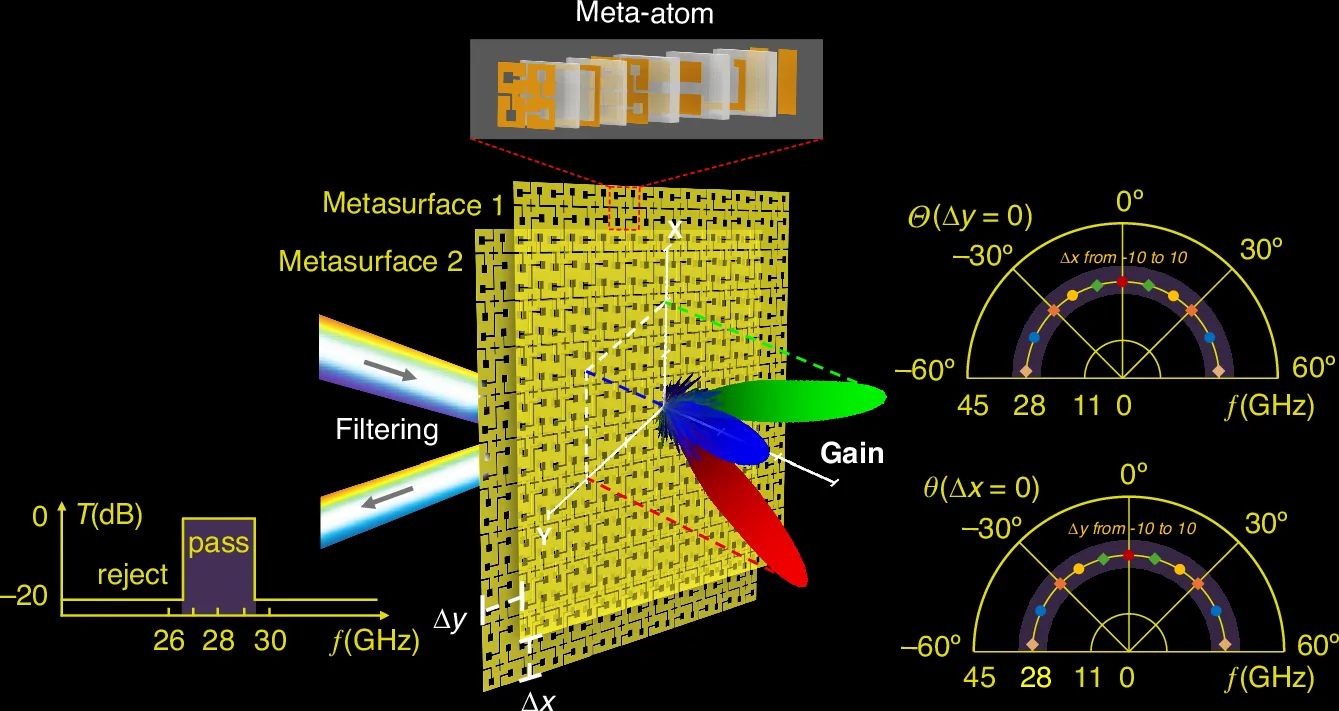
**Illustration of the proposed metasurface for 2-dimensional beam steering and frequency filtering.** The unit cell consists of a multilayered architecture designed to realise both high-order spectral filtering and spatial phase encoding within a single compact structure

Importantly, this large-scale tunability is achieved using an entirely passive platform. Functionality is achieved through controlled displacement between passive structures rather than actively modifying the electromagnetic response of individual meta-atoms. This geometry-driven approach offers a simpler, scalable, and potentially energy-efficient method toward multidimensional control of electromagnetic waves. Looking forward, the material-independent nature of the tuning mechanism suggests that the sliding metasurface concept can be easily extended to other parts of the electromagnetic spectrum, including the terahertz and optical regimes. By combining geometric reconfiguration from sliding metasurfaces with functionalities such as polarisation control, holography, nonlinear frequency conversion, spatiotemporal modulation, and recently developed virtual metasurfaces^14–16^, it is expected to further push the development of future devices that achieve multiple adaptive operations within a single compact architecture. Such capabilities may enable new opportunities for adaptive and intelligent imaging, sensing, and information processing across the electromagnetic spectrum.
